# Diffusion-Weighted MR Imaging in Diagnosis of Superficial and Invasive Urinary Bladder Carcinoma: A Preliminary Prospective Study

**DOI:** 10.1100/tsw.2008.55

**Published:** 2008-04-14

**Authors:** Ahmed El-Assmy, Mohamed E. Abou-El-Ghar, Huda F. Refaie, Tarek El-Diasty

**Affiliations:** ^1^ Urology Department, Urology and Nephrology Center, Mansoura University, Mansoura, Egypt; ^2^ Radiology Department, Urology and Nephrology Center, Mansoura University, Mansoura, Egypt

**Keywords:** MRI, diffusion-weighted MR imaging, urinary bladder carcinoma, apparent diffusion coefficient (ADC) value

## Abstract

We conducted a prospective study to demonstrate the feasibility of using diffusionweighted (DW) magnetic resonance imaging (MRI) for the detection of urinary bladder carcinomas. Between January to June 2007, 43 patients with single bladder tumor were included in our study. Before taking a biopsy, DW MRI was obtained in the axial plane under free breathing scanning with a multisection, spin-echo type, single-shot echo planar sequence with a body coil. Moreover, the apparent diffusion coefficient (ADC) value was measured in a circular region of interest within the carcinoma, urine, normal bladder wall, prostate, and seminal vesicle. All carcinomas in the 43 patients were clearly shown as high signal intensity relative to the surrounding structure. The sensitivity and positive predictive values of DW MRI were 100% in terms of correctly detecting the carcinomas. The ADC value in the carcinoma (1.40 ± 0.51) was significantly lower compared with that of urine (3.50 ± 0.43) (^p^ < 0.001), normal bladder wall (2.29 ± 0.78) (^p^ < 0.001), peripheral zone of prostate (1.77 ± 0.44) (^p^ < 0.05), transition zone of prostate (1.88 ± 0.54) (^p^ < 0.05), and the seminal vesicle (2.12 ± 0.43) (^p^ < 0.001). There was no statistical difference in ADC values between different histological subtypes. There was no overlap between the ADC values of the tumors and the urine, but there was no clear cutoff between the tumor and bladder wall, prostate, or seminal vesicles. Bladder carcinomas have significantly lower ADC when compared to surroundings. Clinical experience with this method is still preliminary and further studies are required.

## INTRODUCTION

Diffusion-weighted (DW) magnetic resonance imaging (MRI) has been a diagnostic tool for a number of years in the field of neuroradiology[1], where DW sequences play an important part in the diagnosis of cerebral ischemia in the hyperacute phase. At the same time, other applications were developed regarding both the characterization of brain tumors and, more recently, the assessment of demyelinating diseases[2].

From applications limited to neuroradiology, steps have progressively been taken in other body regions, such as the abdomen, where it was used in the detection of malignant tumors, such as malignant hepatic, renal, prostatic, colonic, and uterine cervical tumors[3,4,5,6,7,8]. The inherent sensitivity to motion still creates problems in the study of the abdomen due to artifacts caused by the heartbeat and intestinal peristalsis[2].

Therefore, to reduce respiratory motion, the patient must hold his/her breath during the examination[3,4,5,6,7,8]. Such an approach limits the acquisition time, and both the signal-to-noise ratio and the spatial resolution must be compromised as a result. To resolve those problems, Takahara et al.[9] reported a procedure of body DW MRI under free breathing, which enables a longer scan time.

Although motion is an issue with DW imaging in the abdomen, this is much less the case in the pelvis. However, the use of DW MRI under free breathing gives more thin-slice images, with multiple signal averaging, and provides high-quality multiplanar display. Only one study in the literature evaluated the feasibility of DW MRI in the detection of a urinary bladder carcinoma[10]. However, the previous study suffered several limitations.

Our study was conducted to evaluate the feasibility of using DW MRI under free breathing for the diagnosis of a urinary bladder carcinoma. To the best of our knowledge, this is the first single institutional prospective study containing a large number of patients with bladder carcinomas.

## MATERIALS AND METHODS

### Patients

Between January to June 2007, 43 consecutive patients diagnosed to have a bladder tumor were prospectively included in this study. All the patients presented to our outpatient clinic with total hematuria and were evaluated initially with abdominal and pelvic ultrasonography (US), and those with suspicion of bladder tumor were subjected to diagnostic outpatient cystoscopy. Patients with a cystoscopically proven single bladder tumor were subjected to MRI study. After the MRI study, patients were subjected to recystoscopy and biopsy as an inpatient procedure. Our institutional ethical committee reviewed and approved the study protocol. Informed consent was obtained from all patients.

### MR Technique

Before obtaining DW MRI, bladder distension was necessary. Patients were scanned using a 1.5-T MRI scanner (SIGNA Horizon, General Electric Medical Systems, Milwaukee, WI). We started with axial $T_2$-weighted high-resolution MR of the urinary bladder using the following parameters: TR = 7000–8000 msec, TE = 90–102 msec, band width = 20–83 kHz, 256 × 256 matrix, slice thickness of 3 mm, intersection gap of 1 mm, and field of view (FOV) =20 cm.

Then DW MRI was obtained using multisection, fast spin-echo type, echoplanar sequence in axial plane using a body coil. The parameters of image scans were: TR = 8000 msec, TE minimum, band width = 142 kHz, 256 × 256 matrix, slice thickness of 5 mm, intersection gap of 0 mm and FOV = 36 cm, seven excitations, water excitation with *b* value of 0 and 800 sec/mm$^2$. Thirty to 54 slices were obtained in 60–120 sec.

### Image Analysis

The tumor boundaries were selected based on the appearance of tumor on $T_2$-weighted imaging. Image was analyzed using func. tool software (General Electric Medical System, Milwaukee, WI). To nullify interobserver variability, two radiologists (MA, HE), who were blinded to the results of cystoscopy, read all images. They measured the apparent diffusion coefficient (ADC) values of bladder masses, urine, the normal bladder wall, the seminal vesicle, and the transition and peripheral zones of the prostate. To obviate the increased influence of outliers and non-normal distributions, nonparametric tests were used for all hypothesis tests. Comparison of ADC values between carcinomas and surroundings was performed with the Wilcoxon test. The Mann-Whitney U test was used to compare ADC values between histological subtypes. A value of $p < 0.05$ was considered statistically significant.

## RESULTS

The study included 35 males (81.4%) and eight females (18.6%), with mean age 61.3 ± 8.4 years (range, 43–76 years). Tumor appearance was fungating in 15 patients (35.9%), nodular in 16 (37.2%), ulcerative in three (7%), and papillary in nine (20.9%). Tumor length ranged from 2 to 8 cm (mean, 5.1 cm). Histologic findings revealed TCC in 39 patients (90.7%) and SCC in four (9.3%). Muscle invasion by the tumor cells was present in 34 patients (81.4%), while the remaining nine (18.6%) had lamina invasion. Of the patients, 26 (60.5%) were managed by radical cystectomy and urinary diversion, nine (20.9%) by transurethral resection and intravesical immunotherapy, and eight (18.6%) were referred for definitive radiotherapy because of unfitness for cystectomy.

### Imaging Review and ADC Measurements

On the DW images, all carcinomas in the 43 patients were clearly shown as high signal intensity (SI) relative to the surrounding structures (Figs. 1 and 2). There were no false-positive cases. The sensitivity and positive predictive values of DW imaging were 100% in terms of correctly detecting the carcinomas.

The range and[mean and standard deviations] of the ADC values ($\times 10^{-3}$ $mm^2$/sec) were as follows: carcinomas ($n = 43$), 0.57–2.39[1.40 ± 0.51], urine ($n = 43$), 2.81–4.10[3.50 ± 0.43], normal bladder wall ($n = 43$), 0.91–3.89[2.29 ± 0.78.], peripheral zone of prostate ($n = 35$), 0.84–2.7[1.77 ± 0.44], transition zone of prostate ($n = 35$), 0.81–2.90[1.88 ± 0.54], and the seminal vesicle ($n = 35$), 1.24–3.02[2.12 ± 0.43]. ADC values of the carcinomas were significantly lower compared with those of urine ($p < 0.001$), normal bladder wall ($p < 0.001$), prostate ($p < 0.05$), and seminal vesicle ($p < 0.001$). There was no overlap between the ADC values of the tumors and the urine, but in some cases there was an overlap between the ADC values of the tumors and the bladder wall, prostate, or seminal vesicles (Fig. 3). Thus, there seems to be a clear cutoff between the tumors and the urine, but not between the tumors and the bladder wall, prostate, or seminal vesicles. ADC values for histological subtypes were as follows: TCC ($n = 39$), 0.57–3.95[1.60 ± 0.78] and SCC ($n = 4$), 0.87–2.42[1.21 ± 0.24] with no statistical significance ($p = 0.45$).

## DISCUSSION

DW MRI is a technique used to show molecular diffusion, which is the brownian motion of the spins in biologic tissues[11]. The ADC, as a quantitative parameter calculated from the DW MR images, combines the effects of capillary perfusion and water diffusion in the extracellular extravascular space[11]. Thus, DW MRI provides information on perfusion and diffusion simultaneously in any organ, it can be used to differentiate normal and abnormal structures of tissues better, and it might help in the characterization of various abnormalities.

DW MRI is already an established method used routinely at several institutions in the diagnosis of acute stroke[12]. Only in recent years has DW MRI been used in extracranial organs; for example, it has been used both to monitor treatment response and tissue characterization, and to perform functional evaluation of different organs[3,4,5,6,7,8].

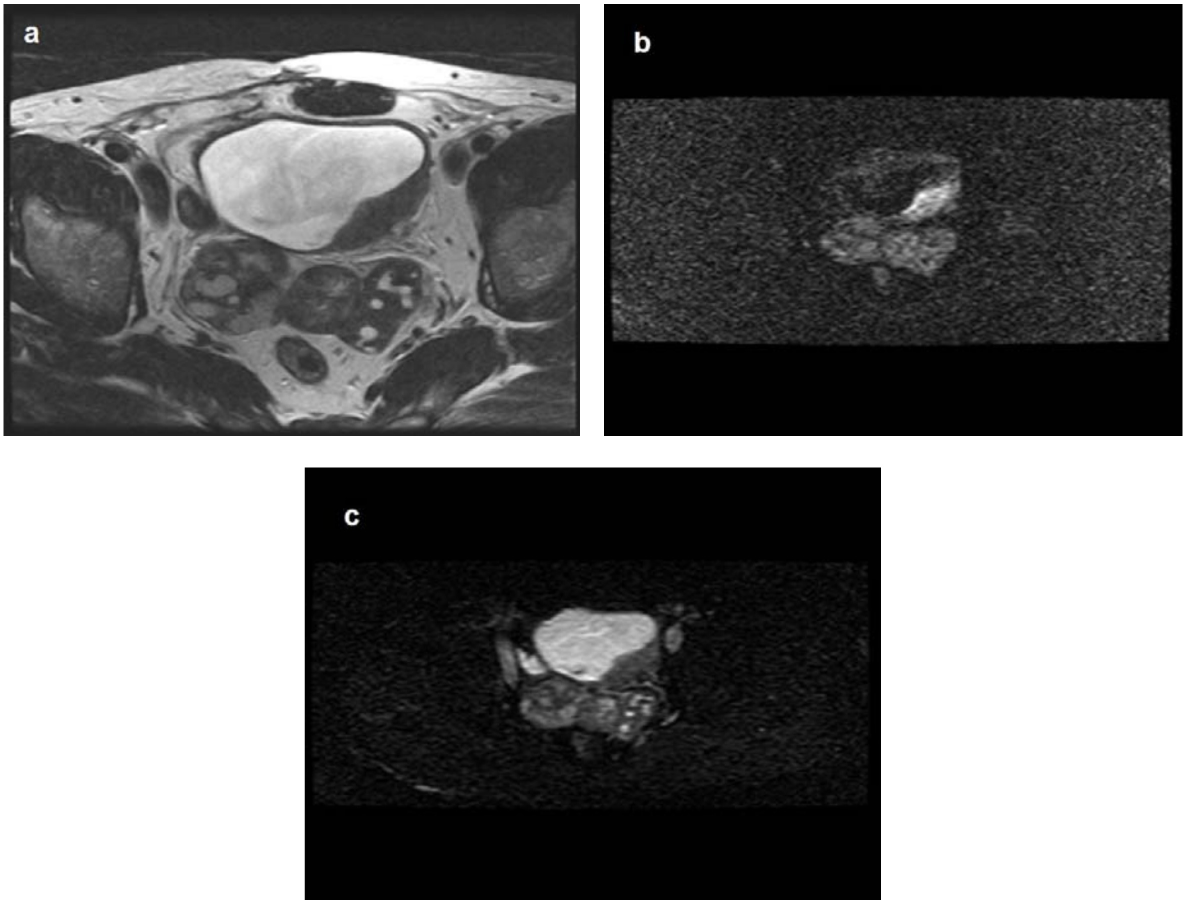

**FIGURE 1**. (a) Axial high-resolution $T_2$-weighted MRI of urinary bladder showing a nodular growth at the posterior aspect of left lateral wall with enlarged seminal vesicles. (b) DW MRI showing the mass with high SI. (c) ADC map showing the mass with low SI.

Previous reports demonstrated that ADC values of malignant hepatic, renal, prostatic, colonic, and uterine cervical tumors were lower than those of benign lesions or normal tissue, and DW MRI showed these malignant tumors as high SI[3,4,5,6,7,8]. The cause of a decrease of the ADC values is considered to be that malignancies commonly have a larger cell diameter and denser cellularity than normal tissue, which restricts water diffusion. However, body DW MRI while the patients hold their breath does not permit thin-slice imaging with adequate signal-to-noise ratio and multiple excitations and, therefore, the clinical use of body DW MRI was limited. Then, Takahara et al.[9] reported body DW MRI under free breathing, which made it possible to obtain more slices with multiple signal averaging, higher signal-to-noise ratio, and high-quality multiplanar display.

The feasibility of using DW MRI under free breathing for the detection of a urinary bladder carcinoma in 15 patients was tested by Matsuki et al.[10]. In their study, all carcinomas were clearly shown as high SI relative to the surrounding structure. The sensitivity and positive predictive values of DW imaging were 100% in terms of correctly detecting the carcinomas. The ADC value of the carcinoma was lower compared with that of the normal bladder wall, the prostate, and the seminal vesicle. However, that study had several limitations; it was a retrospective study assessing few numbers of patients, all patients were examined after the biopsy, and all the tumors were only papillary TCC.

In our study, all patients were evaluated before the biopsy and we included bladder carcinomas of different shapes and histology. All urinary bladder carcinomas showed high SIs and the ADC value of the carcinoma was lower compared with that of urine, the normal bladder wall, the prostate, and the seminal

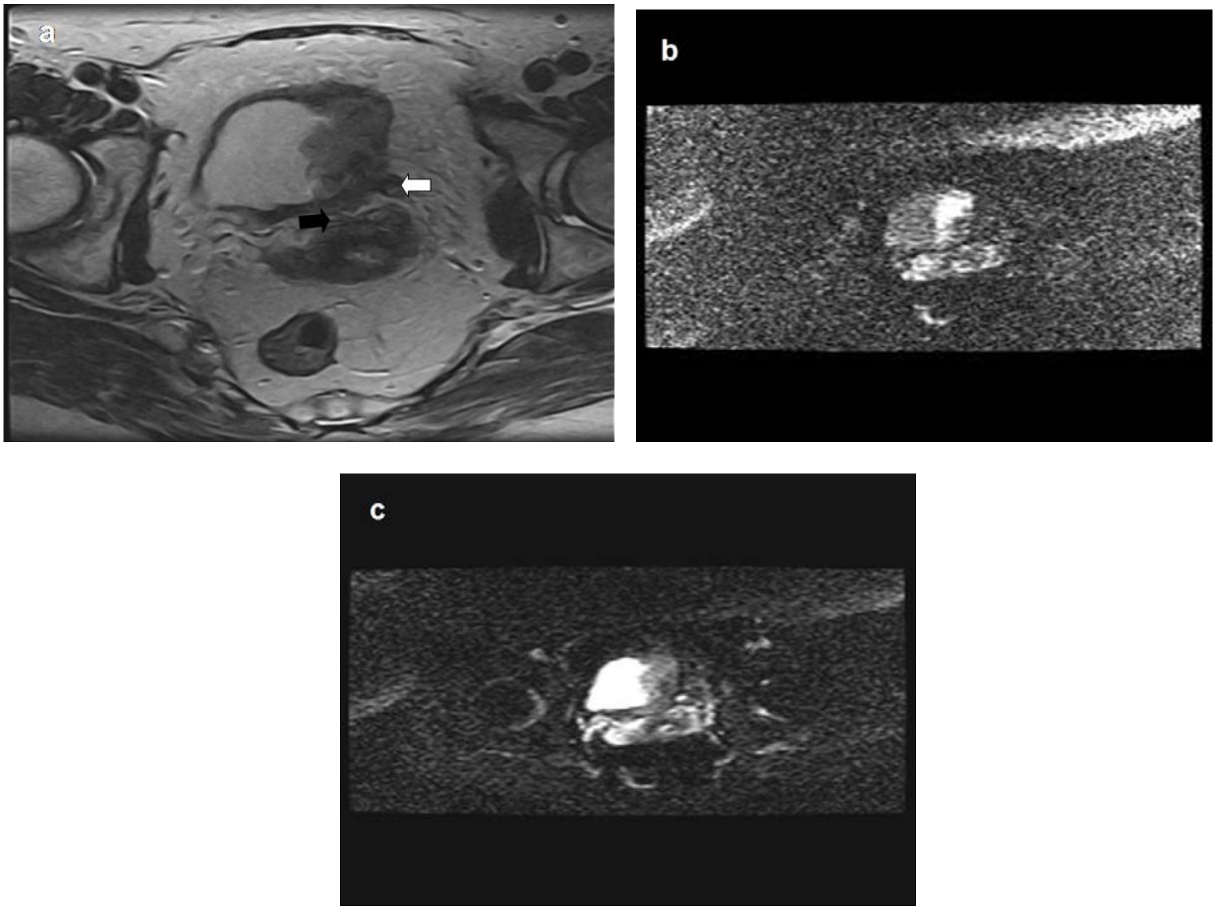

**FIGURE 2.** (a) Axial high-resolution $T_2$-weighted MRI of urinary bladder showing a tumor growth at the left lateral wall infiltrating the left ureter (white arrow) and the corpus uteri (black arrow). (b) DW MRI showing the mass of hyperintense SI with no extravesical extension. (c) ADC map showing the mass with low SI.

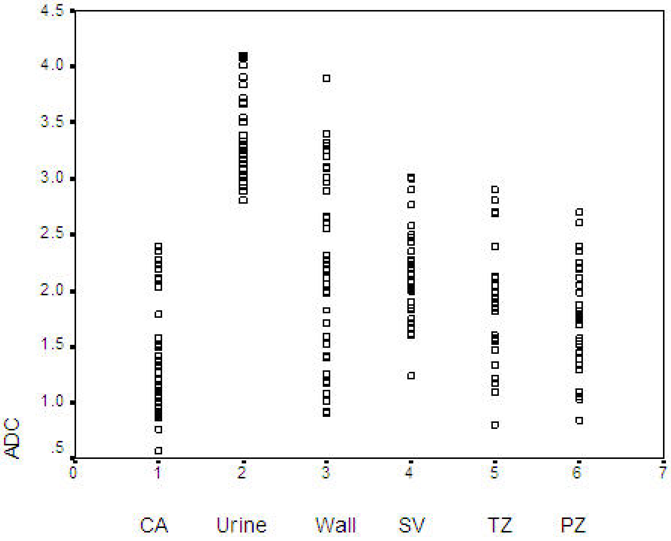

**FIGURE 3.** Scatter-plots of the ADCs of urinary bladder carcinomas (CA), urine, normal bladder wall (Wall), seminal vesicle (SV), transition zone (TZ) of the prostate, and the peripheral zone (PZ) of the prostate vesicle. These results are in harmony to those reported by Matsuki et al.[10]. The sensitivity and positive predictive values of diagnosis bladder carcinoma were 100% similar to previous results[10].

In our study, invasion to the surrounding structures was clearly demonstrated due to high SI of the tumors in comparison to the surroundings. Also there was no overlap between the ADC values of the tumors and the urine, but in some cases there was an overlap between the ADC values of the tumors and prostate or seminal vesicles. These findings are similar to those reported by Matsuki et al.[10]. On the contrary to findings of Matsuki et al.[10], there was an overlap between the ADC values of the tumors and the bladder wall in our study.

We could not find a role for DW MRI in characterizing bladder masses and a correlation, if any, with histological analysis of tumors. However, this finding is questionable because most of our patients had TCC and only four had SCC. Also, there was no inclusion of disease processes that commonly afflict the bladder that are not malignant that could lead to false-positive diagnoses. The sensitivity and positive predictive values of diagnosis bladder carcinoma in our study were 100%; there would also be inherent bias in that the reader would have known that all patients in the study had a bladder carcinoma.

Our study has some limitations. First, most of tumors were large with a mean of 5.1 cm, so it would be critical to assess smaller-size tumors to draw the conclusion of reliability for DW imaging. Second, the effect of viewing images from $T_2$-weighted imaging combined with DW imaging assessed in comparison to blinded review of the DW imaging alone was not assessed; thus, there would be inherent biases in the detection of tumors with DW imaging. Finally, we did not apply this technique to differentiate scars and reactive tissue after the chemotherapy and radiation therapy from the tumor tissue. Further studies are required to evaluate these issues.

## CONCLUSIONS

DW MRI of the urinary bladder seems to be a feasible and reliable method to diagnose bladder carcinoma. Bladder carcinomas have significantly lower ADC when compared to surroundings. This technique could be applied in the clinical area as a rapid addition to existing urinary bladder MRI protocols for diagnosis of bladder carcinoma. However, clinical experience with this method is still preliminary and further studies are required to validate the present results and to better elucidate the clinical application of this technique.
